# IL‐12/15/18‐preactivated NK cells suppress GvHD in a mouse model of mismatched hematopoietic cell transplantation

**DOI:** 10.1002/eji.201445200

**Published:** 2015-04-17

**Authors:** Christian M. Hüber, Jean‐Marc Doisne, Francesco Colucci

**Affiliations:** ^1^ Department of Obstetrics and GynaecologyUniversity of Cambridge School of Clinical Medicine, National Institute for Health Research (NIHR) Cambridge Biomedical Research CentreCambridgeUK

**Keywords:** Cytokine‐induced NK cells, GvHD, HSCT, Lymphoma

## Abstract

Mismatched hematopoietic cell transplants for treating leukemia are complicated by graft versus host disease (GvHD). Here, we show that adoptively transferred IL‐12/15/18‐preactivated NK cells suppress GvHD in a mouse model of fully mismatched hematopoietic cell transplantation. These IL‐12/15/18‐preactivated NK cells maintained Eomesodermin (Eomes) and T‐bet expression upon transfer and, while there was no evidence of direct killing of donor T cells or host DCs by the IL‐12/15/18‐preactivated NK cells, proliferation of donor T cells was inhibited. Strikingly, the graft versus leukemia effect mediated by donor T cells was retained, resulting in improved overall survival of mice that received lymphoma cells, donor allogeneic T cells, and IL‐12/15/18‐preactivated NK cells. These results suggest that IL‐12/15/18‐preactivated NK cells may be useful in improving immunotherapy of mismatched hematopoietic cell transplantation. Compared with previously proposed protocols, our findings suggest that in vitro NK‐cell preactivation with this cytokine cocktail offers the significant advantage that cytokines do not need to be administered systemically to sustain NK‐cell activity, thus avoiding toxicity.

## Introduction

During allogeneic HSC transplantation (HSCT), donor T cells and natural killer (NK) cells cause graft versus leukemia (GvL), which is a beneficial response toward destroying cancer cells. Donor T cells, however, also react against host alloantigens on healthy tissues, causing graft versus host disease (GvHD), which is a potentially lethal condition and must be managed with immunosuppressive therapy. Different immunomodulatory modalities, including immune cells, are being tested in order to improve the management of GvHD and retain the benefit of GvL. Grafted NK cells can suppress GvHD and improve engraftment of HSCs in both mouse models 1 and in patients 2. They may do so by directly killing activated T cells 3, 4, or by targeting host APCs 2, thereby suppressing donor T‐cell proliferation. Importantly, NK‐cell‐mediated suppression of GvHD does not suppress GvL, which is retained 1, 2, 5. One limitation for the use of adoptively transferred NK cells in the clinic is the need for continuous stimulation with exogenous cytokines (i.e. IL‐2), which sustain NK‐cell activity, but cause unwanted toxicity. In order to avoid the toxicity of cytokine‐based therapies, strategies are being designed to preactivate NK cells with cytokines in vitro prior to infusion into patients, thereby eliminating the need to administer cytokines systemically. Adoptively transferred IL‐12/15/18‐preactivated NK cells (also termed cytokine‐induced memory‐like NK cells) carry some of the properties of the recently defined “memory” or “long‐lived” NK cells, in that they proliferate upon adoptive transfer and are long‐lived 6, 7, 8, 9, 10, although they lack antigen‐specific recall responses 11, 12. The ability to mount strong effector functions, alongside their long life span in vivo and the lack of obvious side effects, makes IL‐12/15/18‐preactivated NK cells attractive candidates for cell‐based immunotherapy. Indeed, IL‐12/15/18‐preactivated NK cells have shown efficacy against cancer in mice 9. Using a mouse model of fully mismatched HSCT and lymphoma, we assess here for the first time the ability of NK cells preactivated with either IL‐12/15/18 or IL‐15 alone to suppress acute GvHD without hampering GvL. Our results show that “memory‐like” IL‐12/15/18‐preactivated NK cells suppress GvHD but not GvL.

## Results

### IL‐12/15/18‐preactivated NK cells constitutively produce IFN‐γ and have an activated phenotype

**Figure 1:**
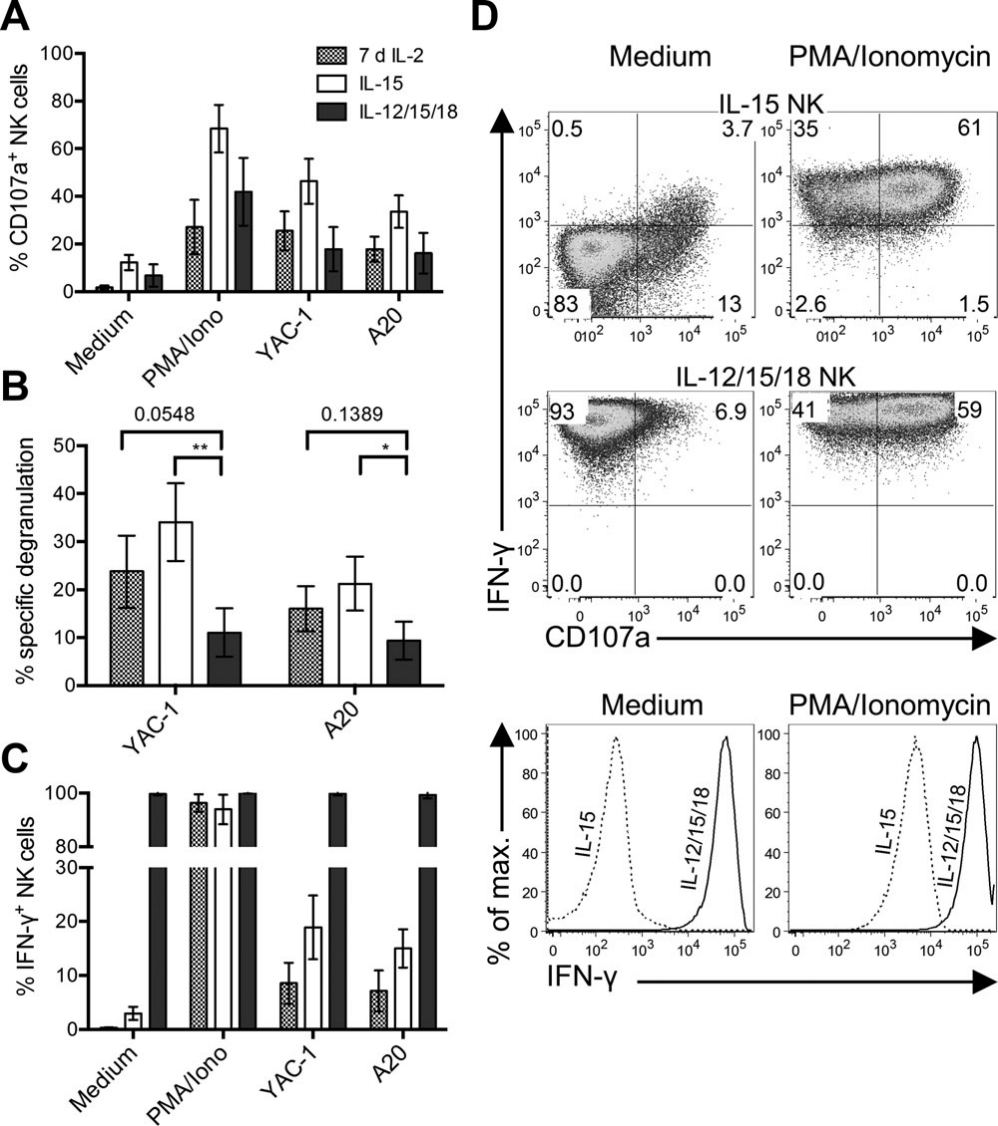
IL‐12/15/18‐preactivated NK cells constitutively produce IFN‐γ. NK cells were purified from the spleens of B6 mice and stimulated for 7 days with IL‐2 (2000 IU/mL) or for 16–18 h with either IL‐15 (10 ng/mL) or IL‐15 + IL‐12 (10 ng/mL) + IL‐18 (50 ng/mL). After careful washing, they were cultured for additional 5 h in medium alone, or stimulated with PMA and ionomycin, YAC‐1 cells, or A20 cells (E:T ratio 1:1). Subsequently, NK cells were stained and analyzed by flow cytometry by gating on living singlet CD3^−^ CD45^+^ NKp46^+^ NK1.1^+^ cells. (A) The percentages of CD107a^+^ NK cells are shown. (B) Specific NK‐cell degranulation (i.e. corrected for background degranulation) upon stimulation with YAC‐1 or A20 tumor cells, from (A). (C) The percentage of IFN‐γ^+^ NK cells from (A) is shown. (A–C) Data are shown as mean ± SD of four samples pooled from four independent experiments with one sample per group. (D) Representative dot plots (top) and histograms (bottom) of IL‐15‐cultured and IL‐12/15/18‐preactivated NK cells cultured in medium alone or activated with PMA and ionomycin, gating on IFN‐γ and CD107a (top) and IFN‐γ (bottom). Statistical analysis was done using one‐way ANOVA followed by Dunnett's multiple comparisons test.

We first quantified both degranulation (measured by expression of extracellular CD107a on NK cells) and intracellular IFN‐γ production by IL‐12/15/18‐preactivated NK cells from C57BL/6 mice (B6, H‐2^b^) upon stimulation with PMA and ionomycin, or in response to T‐cell lymphoma YAC‐1 cells or B‐cell lymphoma A20 cells (Fig. 1A). Specific degranulation was calculated by subtracting background degranulation (percentage of CD107a^+^ NK cells in medium only; Fig. 1B). For comparison we also quantified both degranulation and IFN‐γ production by IL‐15‐preactivated NK cells and NK cells activated with high doses of IL‐2 for 7 days. Activation with high doses of IL‐2 for 7 days is the standard procedure to generate lymphokine‐activated killer (LAK) cells, which mediate potent killing activity. Background degranulation was higher for IL‐12/15/18‐preactivated and IL‐15‐preactivated NK cells (7–13%), than for IL‐2‐activated NK cells (2%). Upon stimulation with tumor cells, specific degranulation of IL‐15‐preactivated NK cells was two‐ to threefold higher (21–34%) than that of IL‐12/15/18‐preactivated NK cells (9–11%). Specific degranulation of IL‐2‐activated NK cells was at an intermediate level (16–24%; Fig. 1A and B). In striking contrast, even in the absence of a stimulus, IL‐12/15/18‐preactivated NK cells were the strongest IFN‐γ producers both in terms of percentage of IFN‐γ positive NK cells (100% of cells, compared to less than 3% among IL‐15‐preactivated and IL‐2‐activated NK cells in the same condition; Fig. 1C) and in terms of IFN‐γ protein per cell, as indicated by the higher fluorescence intensity (Fig. 1D). In essence, we infer that IL‐12/15/18‐preactivated NK cells constitutively produce IFN‐γ as even in medium alone virtually all cells were IFN‐γ^+^.

**Figure 2:**
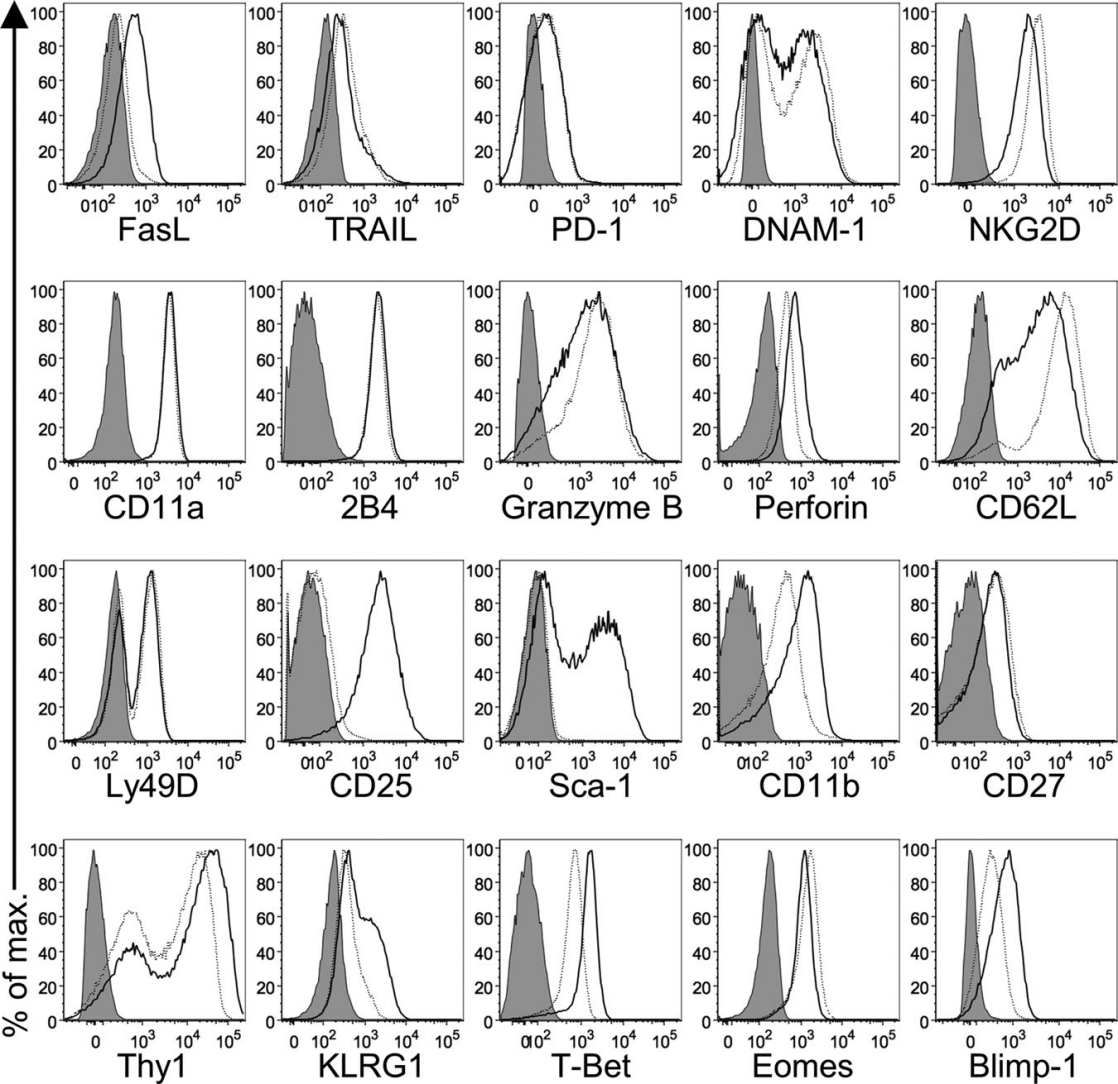
Phenotype of IL‐12/15/18‐preactivated NK cells. NK cells were stimulated with IL‐15 or with IL‐12/15/18 for 16–18 h. They were subsequently washed, stained, and analyzed by flow cytometry by gating on living singlet CD3^−^ CD45^+^ NKp46^+^ cells. The phenotype of IL‐12/15/18‐preactivated NK cells (black line histogram), IL‐15‐cultured NK cells (dotted line histogram), and unstained IL‐12/15/18‐preactivated NK cells as control (gray‐filled histogram) is shown. Histograms are from one experiment representative of *n* = 3 independent experiments, which gave similar results.

We next analyzed the phenotype of IL‐12/15/18‐preactivated NK cells by flow cytometry in comparison to IL‐15‐preactivated NK cells in order to assess the effect of the cytokines on the expression of receptors, effector molecules, transcription factors, and markers of maturity, activation, and differentiation (Fig. 2). IL‐12/15/18‐preactivated NK cells upregulated FasL and Perforin, as well as markers of activation (CD25 and Sca‐1) and maturity (CD11b and KLRG1). CD62L was downregulated, suggesting less homing to LNs but rather to tissues. The transcription factor T‐bet was upregulated, which may explain the sustained IFN‐γ production. However, also Blimp‐1 was upregulated, a transcription factor implicated as a negative regulator of cytokine production in human NK cells 13.

### IL‐12/15/18‐preactivated NK cells suppress acute GvHD and maintain expression of Eomes and T‐bet in vivo

**Figure 3:**
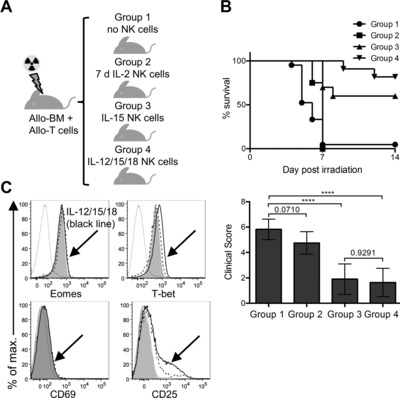
IL‐12/15/18‐preactivated NK cells suppress acute GvHD. (A) The experimental design. BALB/c host mice were lethally irradiated and received myeloprotective T‐cell depleted BMCs from B6 mice. They also received freshly purified splenic allogeneic T cells from B6 mice (basic treatment, Group 1). Mice in Group 2 additionally received IL‐2‐activated NK cells. Mice in Group 3 additionally received IL‐15‐preactivated NK cells, whereas mice in Group 4 additionally received IL‐12/15/18‐preactivated NK cells. (B) Survival curves and clinical scores of mice from the three treatment groups were monitored over time. Statistical analysis of survival was done using log‐rank test; Group 1 versus Group 2: ns (*p* = 0.0817); Group 1 versus Group 3: ***p = 0.0001; Group 1 versus Group 4: ****p < 0.0001; Group 3 versus Group 4: ns (*p* = 0.2146). Clinical scores are shown as mean ± SD of *n* = 8–21 mice per group pooled from at least two independent experiments. Clinical scores were compared applying one‐way ANOVA followed by Tukey's multiple comparisons test for statistical analysis. (C) NK cells were reisolated from spleens of mice in Group 1 and Group 3 at 4 days postirradiation and injection, and analyzed for Eomes, T‐bet, CD69, and CD25 expression by flow cytometry. The phenotype of NK cells from untreated control B6 mice (gray‐filled histogram), non‐NK/NKT cells from untreated control B6 mice (thin dotted line histogram), NK cells from mice in Group 1 (thick dashed line histogram) that are derived from the transplanted BM, and IL‐12/15/18‐preactivated NK cells from mice in Group 3 (thick line histogram) is shown. Histograms are representatives of *n* = 9 mice analyzed in two independent experiments.

Adoptively transferred NK cells improve the outcome of HSCT 1, 2 but are short lived unless stimulated with exogenous cytokines, which cause adverse systemic effects. IL‐15 may cause less severe side effects and it is being explored as alternative to IL‐2. Although IL‐15 may not generate long‐lived NK cells, IL‐12/15/18‐preactivated NK cells are long‐lived in vivo and, upon restimulation, respond vigorously for up to 3 weeks after the first stimulation 6. We reasoned that IL‐12/15/18‐preactivated NK cells, based on their properties, could also improve the outcome of HSCT and may mediate durable and beneficial effects without the toxicity of exogenous cytokines. We therefore set out to address the effect of IL‐12/15/18‐preactivated NK cells from B6 mice on acute GvHD in comparison with both IL‐15‐preactivated NK cells and NK cells activated with IL‐2, using a mouse model of fully mismatched transplantation in which donor allogeneic T cells from the spleens of B6 mice (H‐2^b^) are adoptively transferred to BALB/c mice (H‐2^d^). In this model, BALB/c mice are lethally irradiated and receive myeloprotective T‐cell depleted BM cells from B6 mice. Figure 3A shows a scheme of the experimental setup. Mice in Group 1 received allogeneic T cells and 20 of 21 mice died of acute GvHD within 7 days (Fig. 3B). Mice in Group 2 received IL‐2‐activated NK cells from B6 mice in addition to allogeneic T cells and all eight mice died of acute GvHD within 7 days (Fig. 3B). In striking contrast, only 3 of 10 and none of 11 mice died within 7 days in Group 3 and Group 4, respectively, which received IL‐15‐ or IL‐12/15/18‐preactivated NK cells alongside allogeneic T cells. Indeed, 6 of 10 and 9 of 11 mice in these groups were still alive at day 14 post‐transfer (Fig. 3B). Importantly, the clinical score (denoting severity of acute GvHD) was lowest for both these groups. These results show that both IL‐15‐ and IL‐12/15/18‐preactivated NK cells, but not IL‐2‐activated NK cells, strongly suppress acute GvHD and improve overall survival in our mouse model.

NK cells activated with IL‐2 in vitro do suppress acute GvHD upon adoptive transfer 5, however they also become exhausted and lose expression of Eomesodermin (Eomes) and T‐bet 14. We thus carried out an additional experiment to investigate if IL‐12/15/18‐preactivated NK cells also become exhausted. Mice were treated as in Figure 3A Group 1 and Group 3, and NK cells were reisolated at 4 days after transfer. NK cells present in mice from Group 1 are derived from the transplanted BM cells and can be found in the spleen and other organs of recipient mice. Interestingly, IL‐12/15/18‐preactivated NK cells still expressed Eomes and T‐bet, despite downregulation of the early activation marker CD69 and partial downregulation of CD25 (Fig. 3C) at this time point. This indicated that NK cells preactivated with IL‐12/15/18 do not become exhausted upon adoptive transfer.

### IL‐12/15/18‐preactivated NK cells reduce donor chimerism and inhibit proliferation of donor T cells

**Figure 4:**
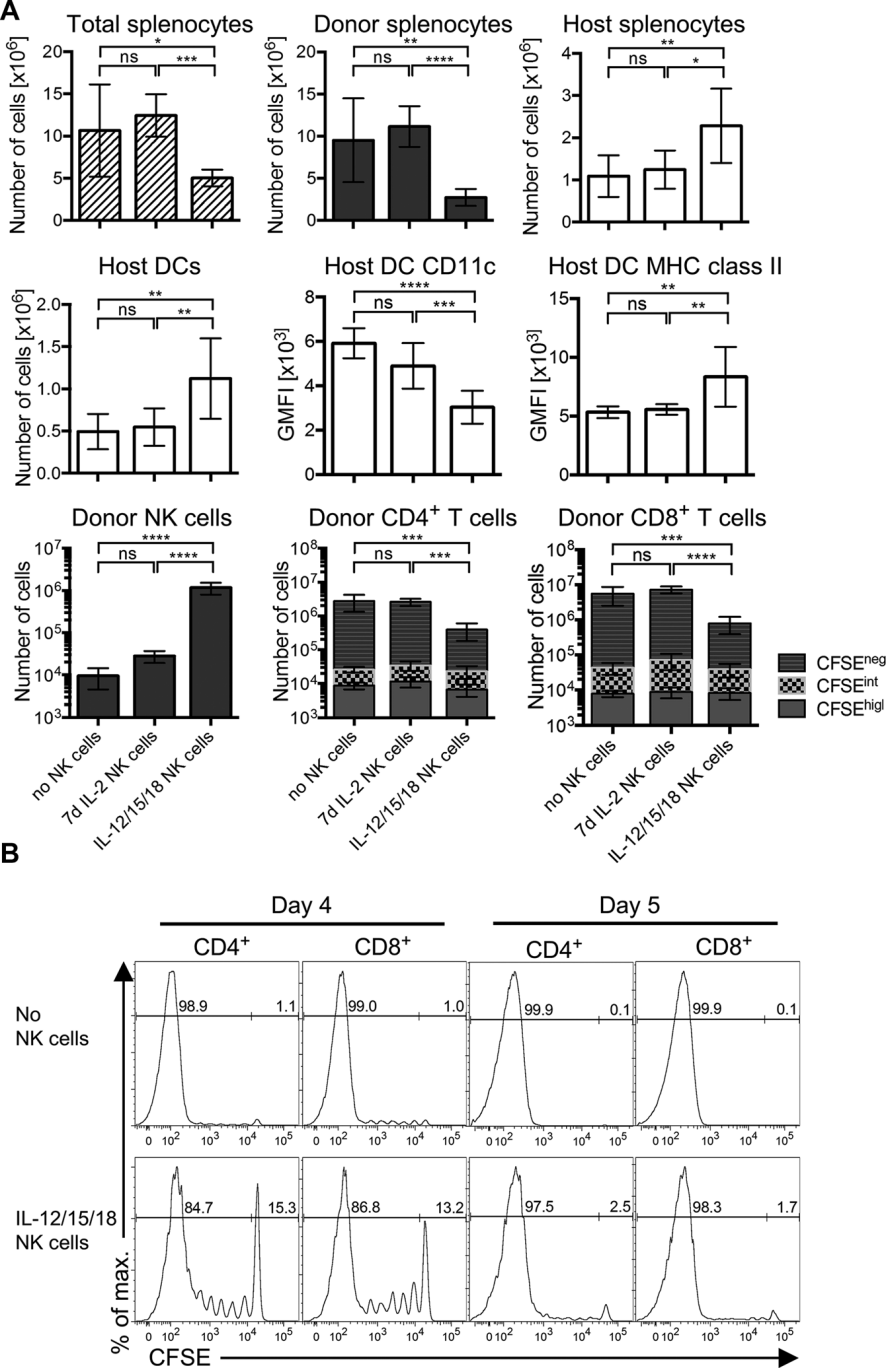
IL‐12/15/18‐preactivated NK cells reduce donor chimerism and inhibit proliferation of donor T cells. Mice were treated as in Fig. 3A, except that in this experiment donor allogeneic T cells were CFSE‐labeled prior to injection. (A) The number of the indicated type of cells per mouse spleen or GMFI (geometric mean fluorescence intensity) on day 5 was determined by flow cytometry. Gating performed on living CD45^+^ singlet cells. Discrimination between host and donor cells was done by H‐2K^b^ (donor)/H‐2K^d^ (host) staining. White bars indicate cells from BALB/c mice (host), whereas dark bars indicate cells from B6 mice (donor). T cells were identified as CD3^+^, NK1.1^−^, and either CD4^+^ or CD8^+^; NK cells were identified as CD3^−^, NKp46^+^, NK1.1^+^; DCs were identified as CD3^−^, NK1.1^−^, CD19^−^, CD11c^+^, I‐A/I‐E^+^. Data are shown as the mean ± SD of *n* = 7–8 mice per group pooled from two independent experiments. Statistical analysis was done using one‐way ANOVA followed by Tukey's multiple comparisons test. (B) CFSE dilution of donor CD4^+^ or CD8^+^ T cells for mice that did not receive NK cells (top) or received IL‐12/15/18‐preactivated NK cells (bottom) was analyzed by flow cytometry 4 or 5 days postirradiation and injection. Histograms are representative for *n* = 7–9 mice/group pooled from two independent experiments for each of the two time points.

To investigate the mechanisms by which IL‐12/15/18‐preactivated NK cells suppress GvHD, we quantified cell numbers in the spleens of mice on day 5 after irradiation and transfer, using the same model as in Figure 3. Thus, one group of mice received no NK cells, one group received IL‐2‐activated NK cells, and the last group received IL‐12/15/18‐preactivated NK cells. All mice also received allogeneic T cells from B6 mice. On day 5 postirradiation and transfer, the total number of splenocytes was approximately half in those mice receiving IL‐12/15/18‐preactivated NK cells, when compared to total splenocyte numbers in mice receiving either no NK cells or IL‐2‐activated NK cells (Fig. 4A). The reduction in total cell numbers was due to lower numbers of H‐2K^b+^ donor cells, which were four‐ to fivefold reduced compared to host mice that received no NK cells or IL‐2‐activated NK cells (Fig. 4A). On the other hand, the number of H‐2K^d+^ host cells in mice receiving IL‐12/15/18‐preactivated NK cells was about twofold greater than in mice receiving either no NK cells or IL‐2‐activated NK cells. Therefore, we infer that adoptively transferred IL‐12/15/18‐preactivated NK cells lower the donor chimerism and preserve host cells.

Grafted donor cells are able to kill allogeneic host cells, due to allorecognition. Host DCs present alloantigens to donor T cells, which causes donor T‐cell activation and proliferation. A lower number of host DCs normally leads to reduced alloactivation of donor T cells and may therefore reduce the GvHD effect. Since mice that received IL‐12/15/18‐preactivated NK cells developed a milder form of GvHD, we hypothesized that IL‐12/15/18‐preactivated NK cells may kill host DCs. For that reason, we measured numbers and activation of host DCs in mice from each of the three treatment groups. Contrary to our hypothesis, we noticed that mice receiving IL‐12/15/18‐preactivated NK cells had an approximately twofold increase in the number of host DCs, which expressed lower levels of CD11c and higher levels of MHC class II molecules (Fig. 4A). In these mice, the number of donor NK cells recovered was over 40‐fold higher (>10^6^ IL‐12/15/18‐preactivated NK cells recovered per spleen) than in mice receiving IL‐2‐activated NK cells (Fig. 4A). Donor T cells (both CD4^+^ and CD8^+^) were reduced by 85–90% in mice receiving IL‐12/15/18‐preactivated NK cells compared to mice receiving IL‐2‐activated NK cells (Fig. 4A). Analysis of CFSE dilution in donor T cells indicated that T‐cell proliferation was strongly reduced in the presence of IL‐12/15/18‐preactivated NK cells. Indeed, the proportion of undivided CD8^+^ and CD4^+^ T cells was strongly increased on day 4 and day 5 in mice receiving IL‐12/15/18‐preactivated NK cells, compared to mice that did not receive NK cells (Fig. 4B). Upregulation of CD25 on T cells ex vivo at day 4 post‐transplant was strongly reduced in mice receiving IL‐12/15/18‐preactivated NK cells (Supporting Information Fig. 1). Additionally, the number of Treg cells was significantly reduced in mice that received IL‐12/15/18‐preactivated NK cells (Supporting Information Fig. 2). This indicates that the reduction in acute GvHD severity observed in mice with IL‐12/15/18‐preactivated NK cells is not due to an increased number of Treg cells.

Collectively, these results suggest that IL‐12/15/18‐preactivated NK cells do not kill host DCs, but may rather promote their activation, presumably through the abundant production of IFN‐γ. On the other hand, IL‐12/15/18‐preactivated NK cells inhibit proliferation and activation of donor T cells. This could contribute to the observed suppression of acute GvHD mediated by IL‐12/15/18‐preactivated NK cells. Another mechanism by which IL‐12/15/18‐preactivated NK cells may suppress acute GvHD could be via direct killing of donor T cells, which become activated by host alloantigens.

### IL‐12/15/18‐preactivated NK cells do not degranulate in response to allogeneic host or syngeneic donor cells

**Figure 5:**
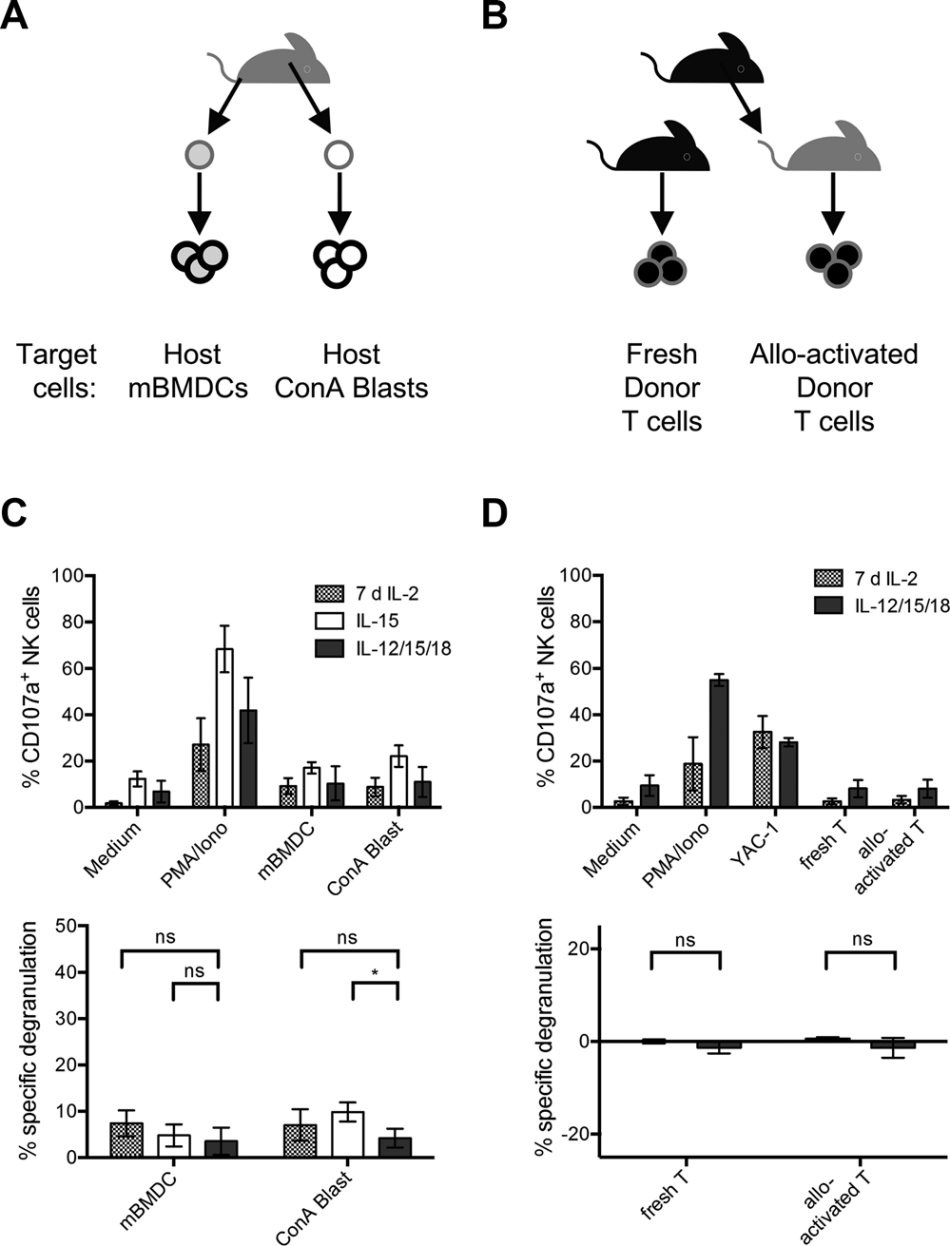
NK‐cell degranulation upon stimulation with allogeneic or syngeneic target cells. NK cells from B6 mice were purified and stimulated as described in Fig. 1 and used as effector cells in a degranulation assay. NK cells were analyzed by flow cytometry and gated on living singlet CD3^−^, CD45^+^, NKp46^+^, NK1.1^+^, H‐2K^b+^ cells. (A, B) The generation of target cells is shown. (A) Allogeneic target cells from BALB/c mice. BM cells were isolated from BALB/c mice, differentiated with GM‐CSF, and maturated with LPS in vitro. ConA blasts were generated by activating BALB/c splenocytes with ConA. (B) Syngeneic target cells from B6 mice. B6 T cells were freshly purified and used as control target cells. Alternatively, they were injected into lethally irradiated BALB/c host mice, causing them to expand due to alloactivation. BALB/c host mice were culled after 5 days and alloactivated donor T cells were purified. (C) The percentage of CD107a^+^ NK cells (i.e. degranulating; top) and specific degranulation (i.e. corrected for degranulation in absence of stimulus; bottom) for IL‐2‐activated NK cells or IL‐15‐preactivated or IL‐12/15/18‐preactivated NK cells stimulated with the indicated stimuli. Data are shown as mean ± SD of *n* = 4 independent experiments with one sample per group and experiment. Statistical analysis was done using one‐way ANOVA followed by Dunnett's multiple comparisons test. (D) Performed as in (C), but with fresh T cells or alloactivated T cells as target cells. Data are shown as mean ± SD of *n* = 3 independent experiments with one sample per group and experiment. Statistical analysis was done using unpaired two‐tailed *t*‐test.

We directly measured degranulation of IL‐12/15/18‐preactivated NK cells upon stimulation with allogeneic target cells and compared it to degranulation of IL‐15‐preactivated NK cells and of NK cells activated with high doses of IL‐2 for 7 days. Target cells were either LPS‐matured BMDCs or splenocytes stimulated with concanavalin A (ConA), which contain mostly activated T cells (Fig. 5A). Both target cells were allogeneic (from BALB/c mice). Upon stimulation with either allogeneic BMDCs or allogeneic ConA blasts, specific degranulation was <10%, irrespective of whether cells were preactivated with IL‐12/15/18, IL‐15, or activated with IL‐2 (Fig. 5C). These results confirm that allogeneic cells do not trigger degranulation in IL‐12/15/18‐preactivated NK cells, which most likely do not kill host cells in vivo.

We show in Figure 4B that IL‐12/15/18‐preactivated NK cells inhibit proliferation of donor T cells in vivo. In order to test the ability of IL‐12/15/18‐preactivated NK cells to kill donor T cells, we activated donor T cells in vivo by transferring splenic T cells from B6 mice into lethally irradiated allogeneic BALB/c mice. After 5 days, we explanted donor T cells from the spleen of host mice and tested the ability of these T cells, previously activated in vivo by alloantigens, to stimulate degranulation in syngeneic IL‐12/15/18‐preactivated NK cells and in IL‐2‐activated NK cells (Fig. 5B). As control targets, we also used unstimulated naïve T cells from the spleens of B6 mice. There was essentially no specific degranulation in response to either naïve T cells or to in vivo activated T cells either by IL‐12/15/18‐preactivated NK cells or by IL‐2‐activated NK cells (Fig. 5D). Taken together, these results indicate that IL‐12/15/18‐preactivated NK cells do not directly kill alloactivated T cells. Indeed, no sign of cell death was observed in either naïve or activated T cells after the 5‐h assay period (data not shown).

### Suppression of GvHD by IL‐12/15/18‐preactivated NK cells is compatible with effective GvL

Donor CD8^+^ T cells kill host tumor cells and are the main mediators of GvL 15. IL‐12/15/18‐preactivated NK cells have been shown to reduce the growth of solid tumors in mice, when combined with radiotherapy 9. However, in our model of lymphoma, no effect on survival was observed when lymphoma‐bearing mice were treated with IL‐12/15/18‐preactivated NK cells (Supporting Information Fig. 3A), suggesting that IL‐12/15/18‐preactivated NK cells are not able to mediate GvL in our model. However, when tumor cells and IL‐12/15/18‐preactivated NK cells were simultaneously coinjected, the IL‐12/15/18‐preactivated NK cells significantly delayed progression (*p* < 0.0001), although tumors were not cleared and all mice died 1.5 days (median) after the control group (Supporting Information Fig. 3B).

**Figure 6:**
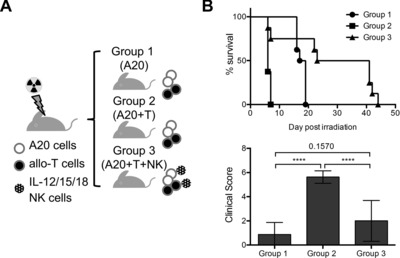
IL‐12/15/18‐preactivated NK cells in GvHD and GvL. (A) The experimental design. BALB/c host mice were lethally irradiated. All mice received myeloprotective T‐cell depleted allogeneic BM cells from B6 mice plus syngeneic A20 lymphoma cells. Mice in Groups 2 and 3 also received splenic T cells from B6 mice. Mice in Group 3 additionally received IL‐12/15/18‐preactivated NK cells from B6 mice. (B) Survival over time and clinical scores shown as mean ± SD of mice from the three treatment groups with *n* = 8 mice per group from two independent experiments. Statistical analysis of survival using log‐rank test; Group 1 versus Group 2: ****p < 0.0001; Group 1 versus Group 3: *p = 0.0240; Group 2 versus Group 3: **p = 0.0023. Clinical scores were compared applying one‐way ANOVA followed by Tukey's multiple comparisons test for statistical analysis.

The ability of IL‐12/15/18‐preactivated NK cells to inhibit donor T‐cell proliferation (Fig. 4) could interfere with T‐cell‐mediated GvL. To test this directly, we used the same model of fully mismatched transplantation as in Figure 3A but this time also administered A20 lymphoma cells (Fig. 6A). Mice that received A20 cells but no allogeneic T cells developed tumors in spleen and liver and all (8/8) died within 19 days (Group 1, Fig. 6B). All mice (8/8) that received A20 cells and allogeneic donor T cells succumbed to acute GvHD within 7 days (Group 2, Fig. 6B). In contrast, mice that received A20 cells, allogeneic T cells, and IL‐12/15/18‐preactivated NK cells survived significantly longer (Group 3, Fig. 6B). In this group, the clinical score for GvHD ranged from 0 to 4 and was therefore much lower than the clinical score of 5–6 for mice in Group 2. Strikingly, the remaining six of eight mice all survived the critical phase of days 15–19, when none of the eight mice in Group 1 survived due to the A20 tumors. This result clearly shows that IL‐12/15/18‐preactivated NK cells suppress GvHD but not GvL. Indeed, we found no remaining tumor cells at the postmortem analysis (data not shown). The surviving six mice, however, had developed late weight loss, reduced activity, and pallor by 40 days and the cellularity of the BM was very low (around 10^5^ cells per femur and tibia combined; data not shown). These signs are compatible with chronic GvHD and with BM failure due to suboptimal engraftment.

## Discussion

We show here that IL‐12/15/18‐preactivated NK cells sustain expression of Eomes and T‐bet and suppress acute GVHD but not GvL in a mouse model of fully mismatched HSCT and lymphoma. We also show that IL‐2‐activated NK cells do not have a significant impact on acute GvHD or on survival in our model, which is in line with previous findings that IL‐2‐activated NK cells become anergic due to downregulation of Eomes and T‐bet 1, 14. The sustained expression of Eomes and T‐bet in IL‐12/15/18‐preactivated NK cells may explain their proliferative potential upon transfer into mice. Indeed, in mice receiving IL‐12/15/18‐preactivated NK cells, the number of NK cells recovered 5 days after transfer was far higher than in mice that had received IL‐2‐activated NK cells. The numbers of IL‐12/15/18‐preactivated NK cells recovered in the spleen alone were greater than the 10^6^ cells injected, indicating strong proliferation. Although we did not analyze long‐term survival of these NK cells, the fact that acute GvHD was strongly inhibited and that mice survived for several weeks indicates that IL‐12/15/18‐preactivated NK cells may persist for longer periods. This is in line with a previous report showing that IL‐12/15/18‐preactivated NK cells are long‐lived 6.

NK cells have been implicated before in the suppression of GvHD by two mechanisms: either by direct killing of donor T cells, which are activated by alloantigens and may upregulate ligands for NK‐cell receptors DNAM‐1 and NKG2D 16, 17, or by killing host DCs hence inhibiting donor T‐cell proliferation 2. Similarly, cytokine‐stimulated human CD56^bright^ NK cells efficiently killed activated autologous CD4^+^ T cells in vitro 3. We found that, in our model, IL‐12/15/18‐preactivated NK cells did not kill either host DCs or donor T cells activated in vivo by alloantigens, and neither did they upregulate DNAM‐1 nor NKG2D. Moreover, the donor chimerism was lower in the presence of IL‐12/15/18‐preactivated NK cells and host cells were preserved. The low donor chimerism may reflect low engraftment of donor HSCs. Indeed, mice that survived acute GvHD developed signs of anemia and showed very low BM cell counts at up to 6 weeks after irradiation. It is unclear as to whether this is a sign of suboptimal engraftment or of chronic GvHD.

IL‐12/15/18‐preactivated NK cells upregulated Perforin and FasL in vitro, thus potentially enhancing cytotoxicity, but they did not vigorously degranulate in response to any of the targets tested, including YAC‐1 and A20 lymphomas, LPS‐matured allogeneic BMDCs, ConA‐activated allogeneic T cells, or naïve or alloantigen‐activated syngeneic T cells. Hence, the mechanism by which IL‐12/15/18‐preactivated NK cells suppressed GvHD is neither through killing host DCs nor by directly killing donor T cells. In addition to the poor degranulation of IL‐12/15/18‐preactivated NK cells in response to YAC‐1 and A20 cells, we also found that IL‐12/15/18‐preactivated NK cells are not able to prolong survival of tumor‐bearing mice, although they can significantly delay progression when coinjected with tumor cells. This may be due to IFN‐γ, which was shown to have not only a beneficial impact on acute GvHD, but also direct antitumor effects and to improve the ability of allogeneic donor T cells to kill host tumor cells 18. Ni et al. showed that, in combination with radiotherapy, IL‐12/15/18‐preactivated NK cells reduced tumor growth and prolonged survival of mice with established tumors 9. Given the different tumor models and route of administration, a direct comparison between the two data sets is difficult: we injected A20 lymphoma cells i.v., whereas Ni et al. administered RMA‐S cells subcutaneously and, in another model, they evaluated lung clearance upon i.v. injection of B16‐RAE‐1ε melanoma cells. Interestingly, production of IFN‐γ and not perforin‐mediated killing is the essential NK‐cell effector function to limit lung metastases caused by B16 melanoma cells in mice 19. Moreover, radiotherapy both initiated reduction of tumor growth and induced homing of IL‐12/15/18‐preactivated NK cells to the localized tumors 9, which we could not obtain due to the systemic spread of A20 tumors. Finally, in Ni et al. it was shown that host CD4^+^ T cells were necessary for the antitumor activity of IL‐12/15/18‐preactivated NK cells 9. The absence of host CD4^+^ T cells in lethally irradiated BALB/c mice may thus be another reason why IL‐12/15/18‐preactivated NK cells did not improve survival of tumor‐bearing mice.

IL‐12/15/18‐preactivated NK cells upregulated T‐bet, constitutively produced IFN‐γ and, upon transfer, might activate host DCs, which in turn may drive donor T‐cell proliferation. Although we found evidence of DC activation (CD11c^low^ and MHC class II^high^) by IL‐12/15/18‐preactivated NK cells, donor T‐cell proliferation in vivo was actually inhibited by IL‐12/15/18‐preactivated donor NK cells. The mechanisms by which this inhibition occurred may well contribute to suppressing GvHD, but remains to be defined, although it is likely that upregulation of the high‐affinity receptor for IL‐2 (CD25) on IL‐12/15/18‐preactivated NK cells makes them sequester IL‐2 by which they may outcompete donor T cells 20. In patients suffering from chronic GvHD, treatment with low‐dose IL‐2 enhanced Treg‐cell counts and significantly suppressed clinical signs of chronic GvHD 21. In our mouse model, IL‐12/15/18‐preactivated NK cells may also outcompete Treg cells for IL‐2, and indeed Treg cells were decreased in mice that had received IL‐12/15/18‐preactivated NK cells. However, the reduction in Treg cells did not cause an increased severity of acute GvHD. The protocol explored preclinically here offers the significant advantage of not requiring systemic cytokine administration to sustain the beneficial effects of NK cells. It will be interesting to assess whether the IL‐12/15/18‐preactivated NK cells and low‐dose IL‐2, which is known to enhance Treg cells, might be sequentially combined to suppress both acute and chronic GvHD.

## Materials and methods

### Mice

C57BL/6 (referred to as B6) and BALB/c mice were purchased from Charles River, UK. All mice were female, used at 8–12 weeks of age and housed at the University of Cambridge Central Biomedical Services under pathogen‐free conditions and according to UK Home Office guidelines. Animal studies have been reviewed and approved by the UK Home Office.

### Cell line

A20 cells (BALB/c B cell lymphoma, H‐2^d^), a gift of Professor K. Smith, and YAC‐1 cells, were maintained in RPMI‐1640 medium with stable Glutamine and supplemented with penicillin/streptomycin (all PAA), 10% FBS (Life technologies), and 50 μM β‐mercaptoethanol (Sigma).

### GvHD and GvL

BALB/c host mice were lethally irradiated (two doses of 4.5 Gy, 3 h apart from each other) and 3 h later injected i.v. with 5 × 10^6^ T‐cell‐depleted BMCs from B6 mice. T‐cell‐depleted BMCs are myeloprotective. Where indicated, mice were also injected with 1 × 10^6^ purified splenic T cells from B6 mice, which cause GvHD, and/or 1 × 10^6^ purified and stimulated NK cells (also from B6 spleens). In GvL experiments, mice additionally received 5 × 10^4^ A20 cells (2.5 × 10^4^ in Supporting Information Fig. 3A where injection was 5 days before irradiation). The GvHD clinical score was assessed on day 5 postirradiation and based on mouse weight loss (0–2), posture (0–2), activity (0–2), and fur texture (0–2), giving a maximum clinical score of 8. Mice were culled before reaching 25% weight loss.

### Cell purification and stimulation

Magnetic beads (CD3 Microbead kit, Pan T Cell Isolation kit II, NK cell isolation kit II; Miltenyi Biotec) were used to deplete T cells in BMCs and to purify donor T cells and donor NK cells from spleens (purity > 90%). Where indicated, NK cells were stimulated for 16– 18 h with 2000 IU/mL rhIL‐2 (Proleukin, Novartis), 10 ng/mL rmIL‐15 (PeproTech), or 10 ng/mL rmIL‐12 (PeproTech) + 10 ng/mL IL‐15 + 50 ng/mL rmIL‐18 (MBL) or for 7 days with 2000 IU/mL rhIL‐2. We found that it is important to wash NK cells thoroughly after preactivation, that is at least three times with a large volume of PBS. LPS‐matured BMDCs were generated as previously described 22. ConA blasts were generated by culturing single cell suspensions from BALB/c spleens for 30 min at 37°C, then collecting nonadherent cells. Nonadherent cells were cultured with 5 μg/mL ConA (Sigma) for 48–72 h before used.

### Degranulation assay

Degranulation assays were performed in 96‐well tissue culture treated U‐bottom plates. A total of 2 × 10^5^–3 × 10^5^ NK cells were plated per well and 1 μg/mL PE‐conjugated anti‐CD107a antibody was added. Where indicated, medium alone, target cells (effector:target ratio 1:1), or PMA (100 ng/mL, Sigma) + ionomycin (2 μg/mL, Sigma) were added to a total volume of 200 μL and incubated at 37°C. After 1 h, Brefeldin A (3 μg/mL, eBioscience) and Monensin (5 μM, Sigma) were added. Cells were then cultured for additional 4 h, subsequently pelleted, washed, and stained for analysis by flow cytometry.

### Flow cytometry

Conjugated mAbs anti‐mouse CD45 (30‐F11), CD3ε (17A2), H‐2K^b^ (AF6‐88.5), H‐2K^d^ (SF1‐1.1), NKG2D (CX5), FasL (MFL3), TRAIL (N2B2), DNAM‐1 (TX42.1), Ly49D (4E5), CD25 (PC61), Perforin (eBioOMAK‐D), Sca‐1 (D7), PD‐1 (29F.1A12), 2B4 (eBio244F4), KLRG1 (2F1), CD11b (M1/70), CD11c (N418), CD27 (LG.3A10), Thy1.2 (30‐H12), CD62L (MEL‐14), CD44 (IM7), NKp46 (29A1.4), NK1.1 (PK136), CD4 (RM4‐5), CD8α (53‐6.7), IFN‐γ (XMG1.2), Eomes (Dan11mag), T‐bet (O4‐46), Blimp‐1 (5E7), CD107a (1D4B), Foxp3 (NRRF‐30), CD16/32‐Fc blocking (93), and anti‐human/mouse Granzyme B (GB11) were purchased from BioLegend, eBioscience, and BD Pharmingen. Intracellular staining was done using the Foxp3 staining buffer set (eBioscience). DAPI (Life technologies) or fixable viability dye eFluor 450 or eFluor 506 (eBioscience) was used to exclude dead cells. Sample acquisition was done on a BD LSR Fortessa (BD Biosciences) using BD FACS Diva software (BD Biosciences) and analyzed using FlowJo (Tree Star).

### Statistical analysis

Statistical analysis was done using GraphPad Prism 6 software. Log‐rank test, two‐tailed unpaired *t*‐test, or ordinary one‐ or two‐way ANOVA followed by Tukey's or Dunnett's multiple comparisons test was used to investigate statistical significance, which was defined as *p* < 0.05. Data are expressed as means ± SD. Asterisks indicate level of significance (*p < 0.05, **p < 0.01, ***p < 0.001, ****p < 0.0001).

## Conflict of interest

The authors declare no financial or commercial conflict of interest.

**Abbreviations**
ConA: concanavalin A
Eomes: Eomesodermin
GvHD: GvH disease
GvL: graft versus leukemia
HSCT: HSC transplantation

## Supporting information

As a service to our authors and readers, this journal provides supporting information supplied by the authors. Such materials are peer reviewed and may be re‐organized for online delivery, but are not copy‐edited or typeset. Technical support issues arising from supporting information (other than missing files) should be addressed to the authors.

Fig. S1. Stronger activation of T cells in mice without IL‐12/15/18‐ preactivated NK cells.
Fig. S2. Reduced number of Tregs in mice that received IL‐12/15/18‐preactivated NK cells.
Fig. S3. IL‐12/15/18‐preactivated NK cells do not eradicate A20 B cell lymphoma cells.
Fig. S4. Gating strategy for flow cytometric analysis of NK cells from *in vitro* experiments.
Fig. S5. Gating strategy for flow cytometric analysis of lymphocytes in GvHD experiments.
