# Supplementary material for: IL‐12/15/18‐preactivated NK cells suppress GvHD in a mouse model of mismatched hematopoietic cell transplantation
Source: Eur J Immunol. 2015 Apr 17;45(6):1727–35. doi: 10.1002/eji.201445200 (PMC4687420; doi:10.1002/eji.201445200)
Supplement: Supplementary file 1 — Fig. S1. Stronger activation of T cells in mice without IL‐12/15/18‐ preactivated NK cells. Fig. S2. Reduced number of Tregs in mice that received IL‐12/15/18‐preactivated NK cells. Fig. S3. IL‐12/15/18‐preactivated NK cells do not eradicate A20 B cell lymphoma cells. Fig. S4. Gating strategy for flow cytometric analysis of NK cells from in vitro experiments. Fig. S5. Gating strategy for flow cytometric analysis of lymphocytes in GvHD experiments. [file eji0045-1727-sd1.pdf]

European Journal of Immunology

Supporting Information for

DOI 10.1002/eji.201445200

Christian M. Hüber, Jean-Marc Doisne and Francesco Colucci

IL-12/15/18-preactivated NK cells suppress GvHD in a mouse model of mismatched hematopoietic cell transplantation

© 2015 The Authors. *European Journal of Immunology* published by Wiley-VCH Verlag GmbH & Co. KGaA, Weinheim.

A

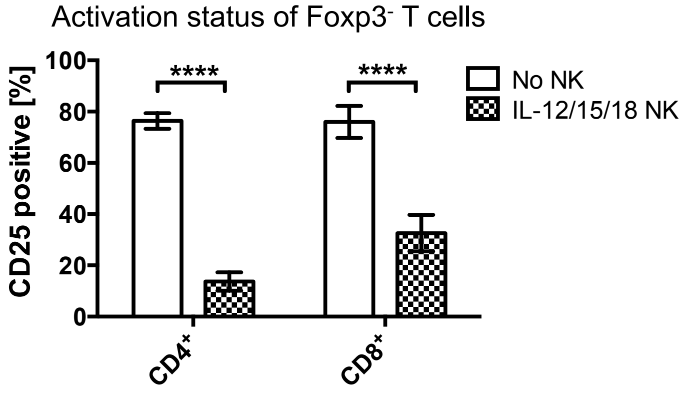

B

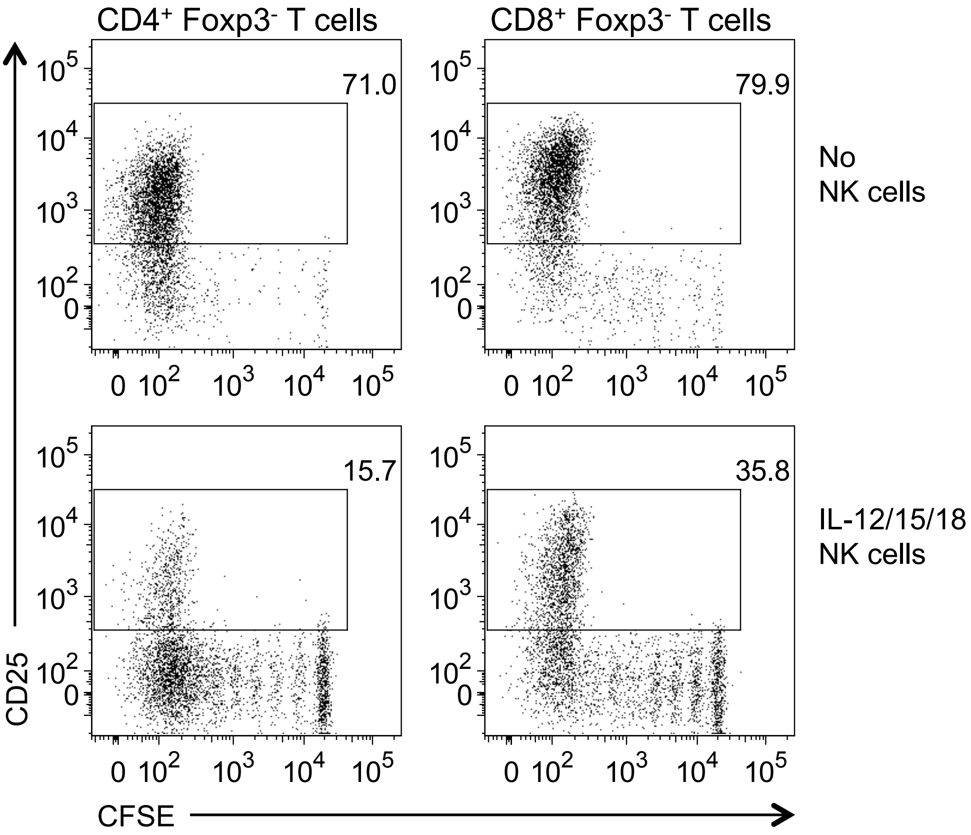

Supp. Fig. 1. Stronger activation of T cells in mice without IL-12/15/18-preactivated NK cells. BALB/c mice were lethally irradiated and injected with allogeneic BMCs with or without IL-12/15/18-preactivated NK cells. Splenocytes were analyzed by flow cytometry after 4 days **A)** CD25 expression of Foxp3⁻ T cells was assessed by flow cytometry. Shown is the mean ± SD of n = 9 mice per group pooled from two independent experiments. Statistical analysis: two-tailed unpaired t-test. **B)** Representative dot plots depicting CFSE dilution and CD25 expression of Foxp3⁻ T cells, which is representative of the 9 mice per group that were analyzed in A).

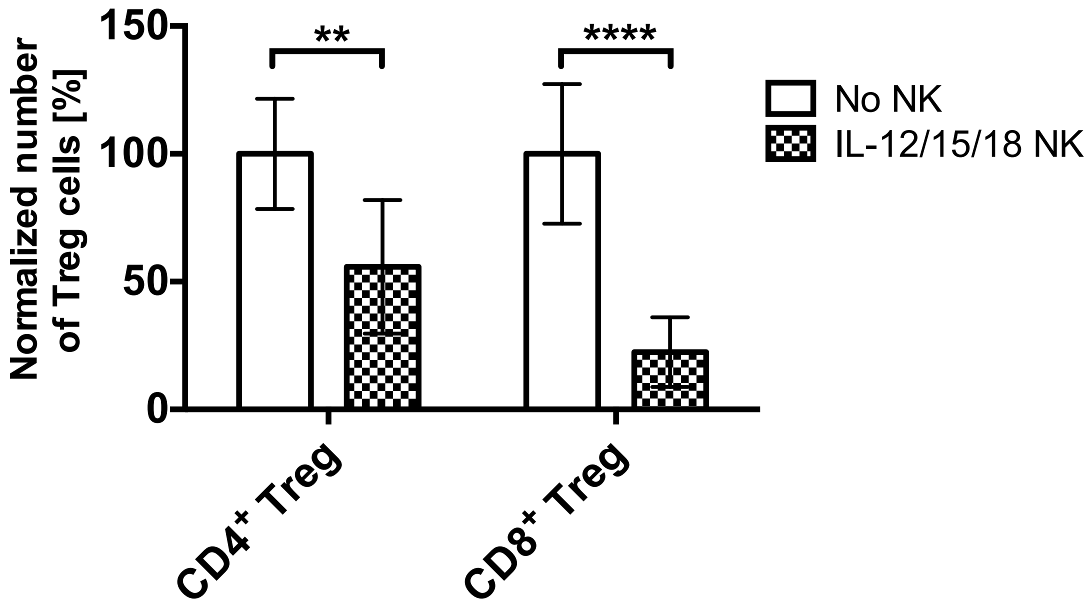

Supp. Fig. 2. Reduced number of Tregs in mice that received IL-12/15/18-preactivated NK cells. BALB/c mice were lethally irradiated and injected with allogeneic BMCs with or without IL-12/15/18-preactivated NK cells. Splenocytes were analyzed by flow cytometry after 4 days. Shown is the normalized number of CD4^{+} or CD8^{+} donor Treg cells (defined as CD25^{+}, Foxp3^{+}, H-2K^{b+}) expressed as the % compared to the control group (BMCs without IL-12/15/18-preactivated NK cells). Shown is the mean ± SD of n = 9 mice per group pooled from two independent experiments. Statistical analysis: two-tailed unpaired t-test.

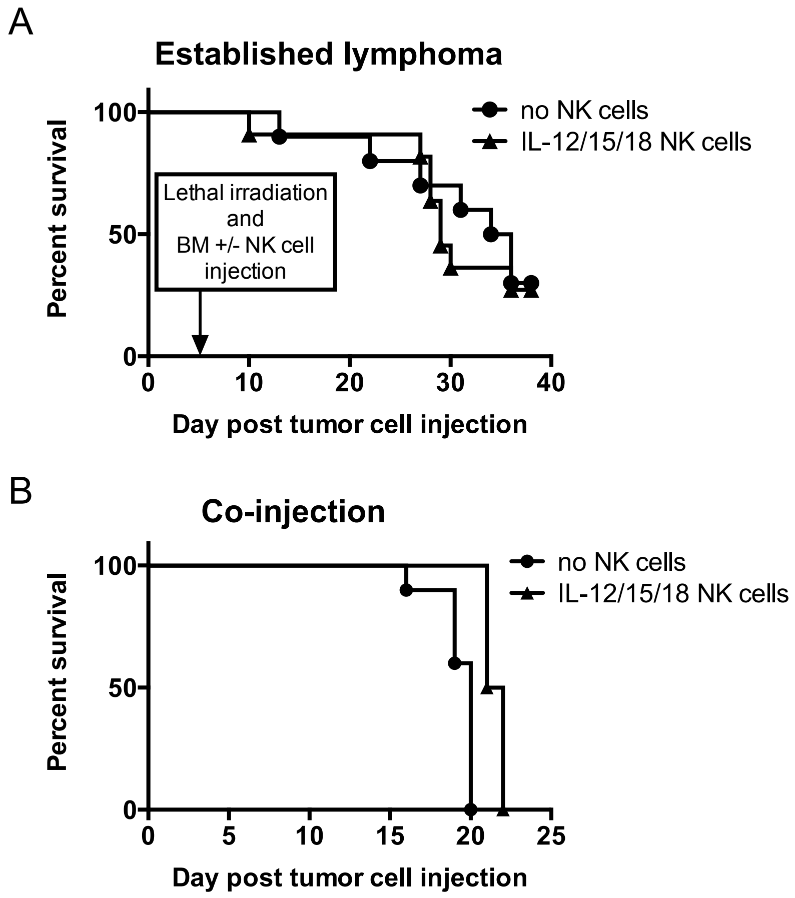

Supp. Fig. 3. IL-12/15/18-preactivated NK cells do not eradicate A20 B cell lymphoma cells. A) BALB/c mice received A20 cells. Mice were lethally irradiated 5 days later and injected with allogeneic BMCs with or without IL-12/15/18-preactivated NK cells. Shown is the survival of n = 10 - 11 mice per group pooled from two independent experiments. The difference between the two groups was not significant (log-rank; ns, $p = 0.7044$). **B)** BALB/C mice were lethally irradiated and injected with A20 cells and allogeneic BMCs with or without IL-12/15/18-preactivated NK cells. Shown is the survival of n = 10 mice per group from two independent experiments. The difference between the two groups was significant (log-rank; **** $p < 0.0001$).

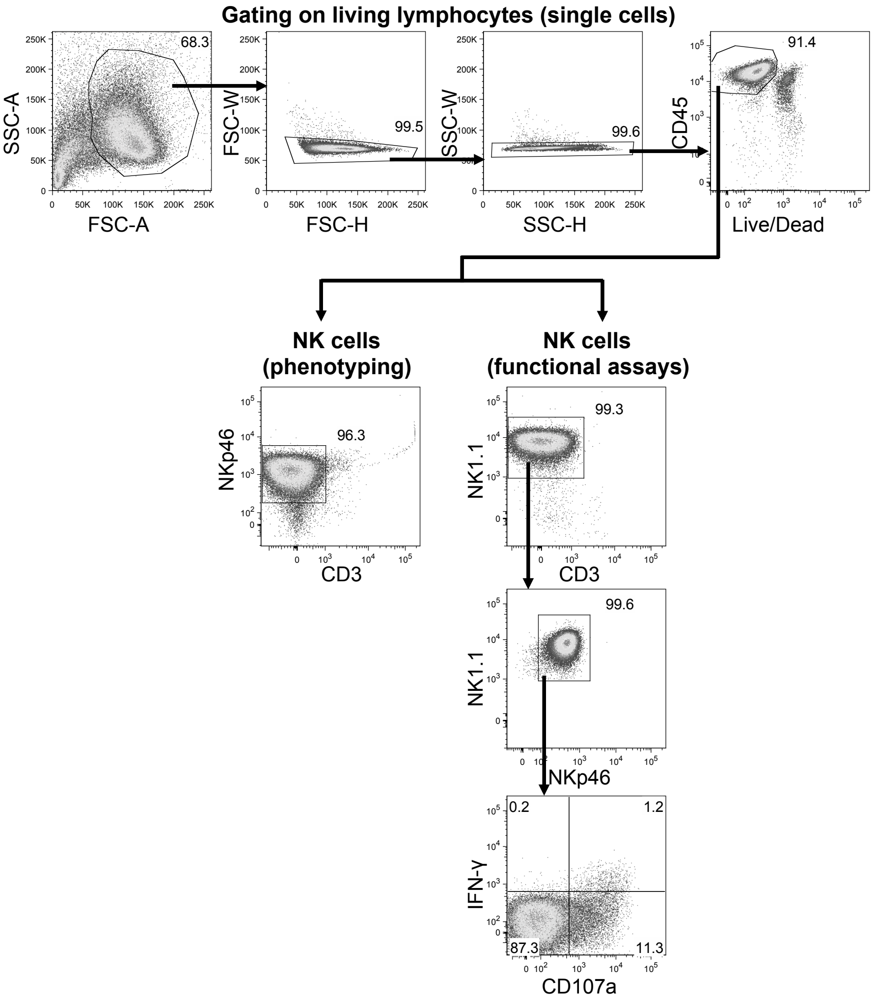

Supp. Fig. 4. Gating strategy for flow cytometric analysis of NK cells from *in vitro* experiments. Gating was done on live single lymphocytes, based on their forward and side scatter, CD45 expression and negative staining for the live/dead marker. Definition of NK cells was based on absence of CD3 expression and positive staining for NKp46 only (phenotyping experiments) or NKp46 and NK1.1 (functional assays).

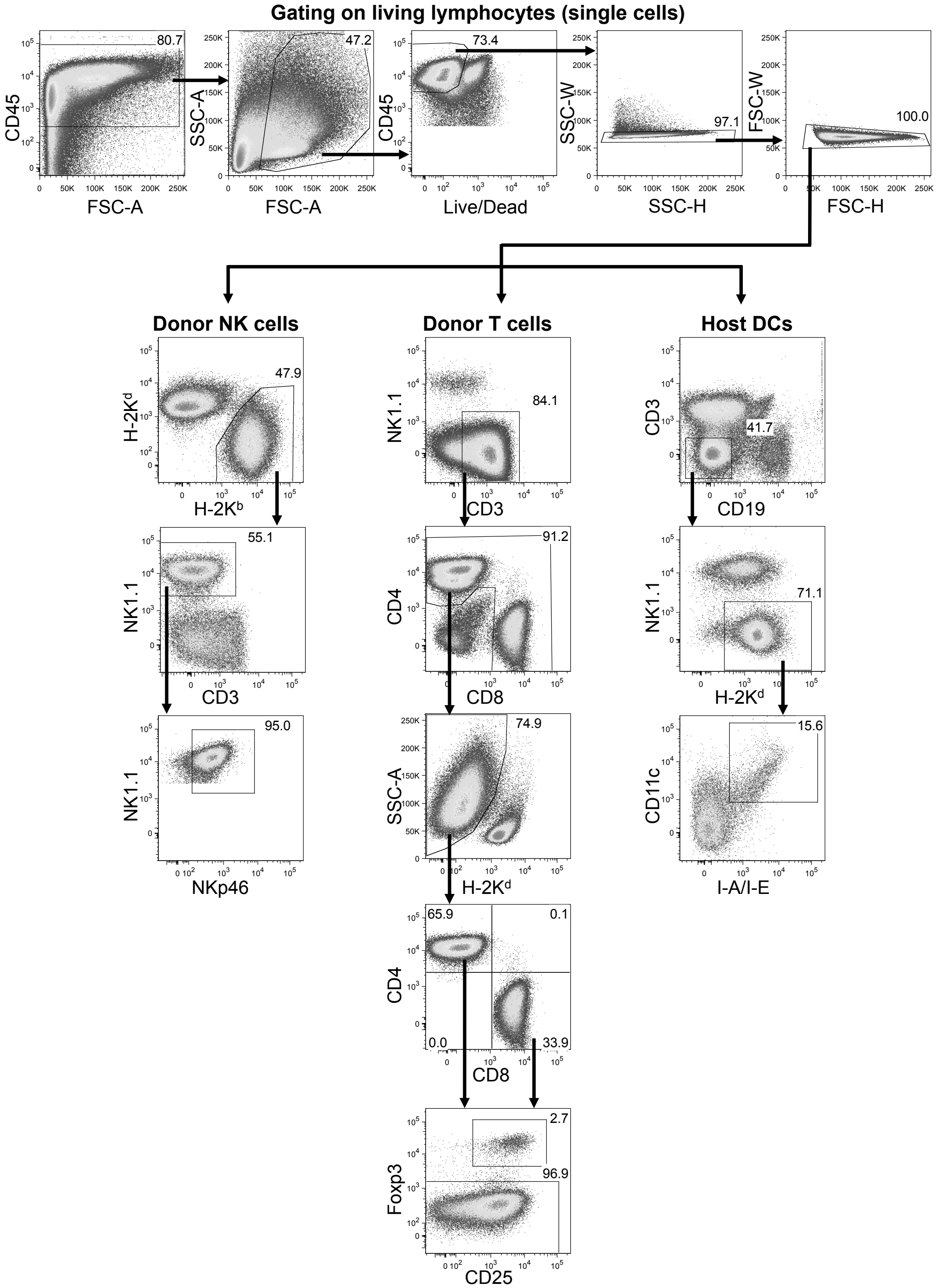

Supp. Fig. 5. Gating strategy for flow cytometric analysis of lymphocytes in GvHD experiments. Due to presence of many debris, dead cells and host cells in irradiated mice, gating slightly differed from *in vitro* experiments. Different subsets of live lymphocytes were defined based on the expression of their surface markers. If not otherwise indicated, cells were defined as follows: a) donor NK cells were defined as H-2K^{b+}, H-2K^{d-}, CD3^{-}, NKp46^{+}, NK1.1^{+}. b) donor T cells were defined as CD3^{+}, NK1.1^{-}, CD4^{+}/CD8^{+}, H-2K^{d-}. Further gating on Foxp3^{+} and CD25^{+} cells defined Treg cells. c) Host DCs were defined as CD3^{-}, CD19^{-}, NK1.1^{-}, H-2K^{d+}, CD11c^{+}, I-A/I-E^{+}.
